# Assessing alternative metrics of methane output measured in a multi-breed, pasture-based sheep population

**DOI:** 10.1093/jas/skag145

**Published:** 2026-05-02

**Authors:** Dermot J Kelly, Nóirín McHugh, Deirdre Purfield, Patrick McCarron, Thierry Pabiou, Craig Murphy, Fiona McGovern

**Affiliations:** Teagasc, Animal and Grassland Research and Innovation Centre, Mellows Campus, Athenry, Co. Galway H65 R718, Ireland; Department of Biological Sciences, Munster Technological University, Bishopstown, Co. Cork T12 P928, Ireland; Teagasc, Animal and Grassland Research and Innovation Centre, Fermoy, Co. Cork P61 P302, Ireland; Department of Biological Sciences, Munster Technological University, Bishopstown, Co. Cork T12 P928, Ireland; Teagasc, Animal and Grassland Research and Innovation Centre, Mellows Campus, Athenry, Co. Galway H65 R718, Ireland; Sheep Ireland, Link Road, Ballincollig, Co. Cork P31 D452, Ireland; Department of Biological Sciences, Munster Technological University, Bishopstown, Co. Cork T12 P928, Ireland; Teagasc, Animal and Grassland Research and Innovation Centre, Mellows Campus, Athenry, Co. Galway H65 R718, Ireland

**Keywords:** methane emissions, sheep, methane metrics, ratio-based traits, repeatability, selection

## Abstract

Reducing methane emissions from ruminant livestock is a global priority, yet no consensus exists on the optimal trait definition for methane emissions. This study compared absolute, ratio-based, and residual methane emission metrics in order to quantify their phenotypic interrelationships and their associations with key production traits in sheep. Gaseous emissions, including methane, and performance data were collected from 15,385 records on 8,182 sheep, including both growing animals and mature ewes, using portable accumulation chambers. Additional data available included live weight, metabolic body weight (MBW), dry matter intake (DMI), slaughter data (carcass weight and days to slaughter), and computed tomography measurements such as rumen volume, predicted kill-out percentage, and kg of muscle/fat mass. Statistical analysis assessed phenotypic correlations, repeatability, and animal ranking differences across methane metrics. The repeatability of methane emissions was moderate (26% in growing animals; 34% in ewes), while body weight was highly repeatable (74% in growing animals; 68% in ewes). Males emitted 0.55–1.36 g/d more than females (*P* < 0.01). Absolute methane emissions were strongly correlated with intensity metrics such as methane per kg of metabolic body weight (0.83 ± 0.01 in growing animals; 0.96 ± 0.004 in ewes), while methane yield (g/kg DMI) was weakly correlated (0.28 ± 0.01 to 0.30 ± 0.01), highlighting its dependence on feed intake variability. Residual methane traits (e.g. residual methane adjusted for metabolic body weight, RMT_MBW_) accounted for performance-related differences and captured individual biological variation (e.g. range −16.6 to 13.4 g/d in ewes), with moderate correlations to absolute methane emissions (*r* = 0.54–0.68). Animals selected for low methane yield or residual traits tended to have higher DMI and daily emissions, while absolute trait selection favored smaller, less productive animals. These findings demonstrate that methane metrics capture distinct biological processes and are not interchangeable. Trait choice must align with breeding and production objectives, whether to reduce total emissions, improve efficiency, or identify inherently low emitters.

## Teaser

This study evaluates alternative metrics for methane emission traits in sheep, exploring how these metrics vary across life stages and with production traits and what each reveals about methane emission patterns in pasture-based systems.

## Introduction

Methane emissions from livestock significantly contribute to global greenhouse gas emissions, posing a critical challenge for sustainable agriculture. Methane, a by-product of enteric fermentation in ruminants, has a global warming potential approximately 80 times greater than carbon dioxide, making it a key target for climate mitigation efforts in livestock farming (Black et al. 2021; Bačėninaitė et al. 2022; Smith et al. 2022). Strategies to reduce methane emissions must prioritize environmental goals whilst remaining cognizant of production needs, particularly given that substantial increases in food production are required to meet future demands (Falcon et al. 2022).

Methane can now be measured in large numbers of individual animals due to the evolution in recording techniques, including the portable accumulation chambers (PAC), a practical and cost-effective method of providing repeatable methane measurements in sheep (O’Connor et al. 2021). The availability of large quantities of methane records coupled with individual performance records has enabled the relationships between methane output and important production traits to be investigated across both cattle and, to a lesser extent, sheep (Herd et al. 2014; Bird-Gardiner et al. 2017; Crowley et al. 2024). Enteric methane emissions have also been shown to be under moderate genetic control in sheep, with heritability estimates ranging from 0.13 to 0.29 (Pinares-Patiño et al. 2013; Jonker et al. 2018). Higher body weight and greater dry matter intake (DMI) have been associated with increased methane production across both cattle and sheep (*r* ∼0.41–0.75) (Bird-Gardiner et al. 2017; Crowley et al. 2024; O’Connor et al. 2024), whereas higher body condition scores (BCS) have been associated with lower methane production (McGovern et al. 2022; Starsmore et al. 2024).

Given these associations between methane and production traits, it is important to consider emission reductions in a holistic sense, balancing overall productivity and efficiency; this has resulted in alternative metrics of methane emissions being proposed (Duthie et al. 2018; Boland et al. 2020; O’ Connor et al. 2021). A plethora of studies have used metrics such as methane per unit of intake (i.e. methane yield) across both cattle and sheep (Goopy et al. 2014; Starsmore et al. 2024; Woodmartin et al. 2024), as well as linking methane to weight or growth (i.e. methane intensity) (Smith et al. 2021; Gebbels et al. 2022; Crowley et al. 2024). However, these traits have often been used descriptively, without systematic evaluation of how alternative definitions of methane relate to one another or to key production traits.

It is unlikely that any single metric can fully capture all aspects of methane efficiency, given the complex interplay of genetics, diet, rumen microbiota, and physiology effecting an animal’s methane emissions (Soren et al. 2017; Zhang et al. 2023). Moreover, an animal’s methane output is not static; it can vary across stages of growth and lactation due to changes in nutrient demands, feed intake, and physiological state (O’Connor et al. 2024).

The objectives of the present study were to firstly derive alternative metrics of methane emissions across a diverse dataset of sheep spanning life-stages, breeds, and pasture-based systems and secondly to quantify the inter-relationship among these alternative metrics and their relationships with key production traits. This research will enhance our understanding of the trade-offs between emissions reduction and productivity while also providing valuable insights for targeted breeding and management strategies.

## Materials and methods

### Ethics

Data were generated on growing animals and ewes from 121 sheep flocks in Ireland. Data collection was approved by the Teagasc Animal Ethics Committee (TAEC2020-252; TAEC2020-258; and TAEC0323-374) and the Health Protection Regulatory Authority (AE19132/P112; AE19132/P114; and AE19132/P181).

### Data

A total of 15,385 measurements of methane output (g d^−1^) were collected from 8,182 sheep across 121 flocks, at a variety of different life-stages, between the years 2019 and 2024 inclusive. The measured production stages included: lambs (105 to 365 d of age), hoggets (nulliparous 2-tooth females), and lactating and dry (non-pregnant and non-lactating) ewes. Animals were categorized as two broad datasets: growing animals and ewes. Growing animals were defined as males and nulliparous females aged between 105 and 600 d of age at the time of measurement (*n* = 4,080 animals). Ewes were defined as females that had lambed at least once before the methane measurement date and were aged between 380 and 3,850 d (*n* = 4,328 animals), some animals appeared in both datasets. The ewe dataset consisted of methane measurements measured during two lactation status categories: 1. during lactation, with lambs at foot prior to weaning and ≤100 d post-partum (*n* = 1,709 records), and 2. during the non-lactating or dry period, when lambs are weaned and prior to mating and > 100 d post-partum (*n* = 4,970 records).

#### Gaseous emissions data

All gaseous emission measurements were captured using a PAC under controlled conditions as described by (O’Connor et al. 2021). Portable accumulation chambers enable high throughput, short term spot sampling of emissions, capturing methane and carbon dioxide concentrations at multiple time points within the measurement period, which are subsequently converted into daily methane and carbon dioxide emission estimates (O’Connor et al. 2021). Animals were randomly assigned to 1 of the 12 PAC chambers. For each measurement session, animals were removed from their diet at least one hour prior to measurement. Each animal was weighed using a Prattley weighing scales (O’ Donovan Engineering Co. LTD, Cork, Ireland) prior to PAC measurement. Once an animal entered the PAC, the door was sealed, and the measurement period commenced. Methane and carbon dioxide output and oxygen consumption data were obtained from the sheep upon entry and after a 50-min measurement period using an RKI Eagle 2 monitor (Weatherall Equipment and Instruments Ltd, UK), a gas detection device inserted through the chamber’s sampling valve. Portable accumulation chamber measurements were collected across multiple research and commercial flock trials conducted over several years, resulting in repeated methane records for a subset of animals (979 growing animals and 1,096 ewes). In some trials, repeat measurements were conducted within short intervals (≤14 d; *n* = 547 animals), while in others, repeat records were separated by longer periods (>14 d; *n* = 1,528 animals).

Within both the growing animals and ewes, gaseous methane records where an animal emitted less than 4 g d^−1^ and records that were ± 3 SD from the daily mean (within respective datasets) were removed (*n* = 186 growing animals and 70 ewes). For all methane measurements, growing animals and ewes were separately assigned to a contemporary group based on date of measurement, flock, and PAC run number (i.e. the 12 animals using the PACs at the same time). Only contemporary groups with at least five animals were retained for all further analysis. Body weight measurement coincided with the methane measurement, and only growing animals with weights ranging between 25 and 80 kg and ewes weighing between 55 and 100 kg were retained. Metabolic body weight (MBW) was calculated for all animals as body weight^0.75^. Following data editing, 6,778 methane records from 3,413 growing animals and 6,679 methane records from 3,941 ewes remained (Tables 1 and 2). Among growing animals these records comprised 4,935 measurements from females and 1,843 from males, stemming from 2,162 female and 1,251 male animals. For the development of carbon dioxide–based traits, carbon dioxide measurements exceeding 2,500 g d^−1^ were excluded, yielding 6,770 valid carbon dioxide observations from 3,409 growing animals and 6,547 observations from 3,900 ewes.

**Table 1:** Number of records, number of animals, mean (μ) and standard deviation (SD; in parenthesis), trait range and coefficient of variation (CV) for production traits and absolute, ratio and residual methane traits in growing animals.

Metric	Trait^1^	No. recs	No. ani	μ (SD)	Range	CV
**Production**	Body weight (kg)	6,778	3,413	47.68 (11.98)	20.00–82.20	25.12
	Metabolic body weight (kg)	6,778	3,413	18.04 (3.39)	9.46–27.30	18.79
	Dry matter intake (k g d^−1^)	180	180	1.53 (0.60)	0.40–2.55	39.28
	Average daily gain (k g d^−1^)	2,459	1,812	0.21 (0.05)	0.15–0.35	23.81
	Carcass weight (kg)	901	901	20.00 (2.14)	15.20–26.00	10.72
	Rumen volume (litres)	756	756	6.56 (1.52)	2.33–12.27	23.17
**Absolute**	Methane (g d^−1^)	6,778	3,413	14.00 (5.15)	4.02–32.26	36.79
**Ratio**	MI_BW_	6,703	3,352	0.30 (0.11)	0.06–0.81	36.67
	MI_MBW_	6,703	3,352	0.79 (0.28)	0.17–1.97	35.44
	MI_ADG_	2,459	1,812	70.17 (25.61)	14.26–184.44	36.50
	MI_CW_	836	836	0.80 (0.29)	0.22—1.69	36.25
	MI_MM_	740	719	1.42 (0.48)	0.33–3.08	33.09
	MI_FM_	735	718	5.93 (2.79)	0.95–22.65	47.05
	MI_KO_	740	718	37.08 (12.001)	8.34–80.22	32.37
	MI_M: F_	734	712	4.35 (2,05)	0.94–16.15	47.12
	MI_Rumen_	734	718	2.70 (0.92)	0.65–6.87	34.02
	MY	573	180	11.05 (5.72)	1.91–40.21	51.76
	CH_4_/(CH_4_ + CO_2_)	6,770	3,409	0.02 (0.002)	0.003–0.19	41.92
	CO_2_ Yield	573	180	616.90 (448.23)	20.05–2853.20	72.66
**Residual**	RMT_BW_ (g d^−1^)	6,568	3,214	0.00 (1.99)	−10.37 to 10.08	NA
	RMT_MBW_ (g d^−1^)	6,568	3,214	0.00 (1.99)	−10.36 to 10.12	NA
	RMT_DMI_ (g d^−1^)	830	177	0.00 (2.45)	−11.06 to 7.63	NA
	RMT_MBW+DMI_ (g d^−1^)	830	177	0.00 (2.38)	−9.96 to 7.78	NA
	RMT_ADG_ (g d^−1^)	2,105	1,536	0.00 (1.85)	−9.27 to 10.81	NA
	RMT_CW_ (g d^−1^)	1,085	657	0.000 (1.96)	−8.64 to 8.00	NA
	RMT_MBW+ADG_ (g d^−1^)	2,105	1,536	0.00 (1.90)	−9.00 to 9.27	NA

1 MI_BW_: methane per kg of body weight, MI_MBW_: methane per kg of metabolic body weight, MI_ADG_: methane per kg of average daily gain, MI_CW_: methane per kg of carcass weight, MI_MM_: methane per kg muscle mass, MI_FM_: methane per kg fat mass, MI_KO_: methane per unit KO%: MI_M: F_: methane per muscle-to-fat ratio, MI_Rumen_: methane per litre of rumen volume, MY: methane yield per kg dry matter intake, CH_4_/(CH_4_ + CO_2_): methane production as a proportion of methane and carbon dioxide production, CO_2_ Yield: carbon dioxide per kg of dry matter intake, RMT_BW_: residual methane adjusted for body weight, RMT_MBW_: residual methane adjusted for metabolic body weight, RMT_DMI_: residual methane adjusted for dry matter intake, RMT_MBW+DMI_: residual methane adjusted for metabolic body weight and dry matter intake, RMT_ADG_: residual methane adjusted for average daily gain, RMT_CW_: residual methane adjusted for carcass weight, RMT_MBW+ADG_: residual methane adjusted for metabolic body weight and average daily gain.

**Table 2:** Number of records, number of animals, mean (μ) and standard deviation (SD; in parenthesis), trait range and coefficient of variation (CV) for production traits and absolute methane, ratio and residual methane traits in ewes.

Metric	Trait^1^	No. recs	No. ani	μ (SD)	Range	CV
**Production**	Body weight (kg)	6,679	3,941	25.40 (2.36)	20.47–31.39	9.29
	Metabolic body weight (kg)	6,679	3,941	74.81 (9.26)	56.00–99.00	12.38
	Dry matter intake (k g d^−1^)	460	270	1.86 (0.72)	0.44–4.13	38.73
**Absolute**	Methane (g d^−1^)	6,679	3,941	21.07 (7.83)	4.01–44.89	37.16
**Ratio**	MI_BW_	6,679	3,941	0.28 (0.11)	0.04–0.68	39.29
	MI_MBW_	6,679	3,941	0.83 (0.31)	0.14–1.89	37.35
	MY	871	272	16.28 (8.11)	2.66–66.44	49.82
	CH_4_/(CO_2_ + CH_4_)	6,547	3,900	0.02 (0.005)	0.003–0.13	30.83
	CO_2_ Yield	850	267	993.91 (474.23)	150.66–4199.10	47.71
**Residual**	RMT_BW_ (g d^−1^)	6,517	3,801	0.00 (3.07)	−16.62 to 13.46	NA
	RMT_MBW_ (g d^−1^)	6,517	3,801	0.00 (3.07)	−16.60 to 13.45	NA
	RMT_DMI_ (g d^−1^)	803	269	0.00 (3.29)	−13.30 to 9.94	NA
	RMT_MBW+DMI_ (g d^−1^)	803	269	0.00 (3.25)	−13.58 to 9.59	NA

1 MI_BW_: methane per kg of body weight, MI_MBW_: methane per kg of metabolic body weight, MY: methane yield per kg dry matter intake, CH_4_/(CH_4_ + CO_2_): methane production as a proportion of methane and carbon dioxide production, CO_2_ Yield: carbon dioxide per kg of dry matter intake, RMT_BW_: residual methane adjusted for body weight, RMT_MBW_: residual methane adjusted for metabolic body weight, RMT_DMI_: residual methane adjusted for dry matter intake, RMT_MBW+DMI_: residual methane adjusted for metabolic body weight and dry matter intake.

All animals with a methane phenotype formed part of either research flocks or flocks participating in the Irish national sheep breeding programme (www.sheep.ie), therefore additional detailed phenotypes were available on animals, including: feed intake, slaughter data, growth rates, computed tomography (CT) scans, and lambing records. Information was also available on each animal’s birth and rearing litter size, as well as breed and heterosis and recombination loss. Breed composition data were available for all animals on the main breeds represented in the dataset, which included Belclare, Blackfaced Mountain, Charollais, Cheviot, Lleyn, Suffolk, Texel, and Vendeen. Heterosis and recombination loss coefficients were calculated for each animal as: $1-\sum_{i=1}^{N}{\mathrm{sire}}_{i}\;.\;{\mathrm{dam}}_{i}$ (VanRaden 1992) and $1-\sum_{i=1}^{N}\frac{{\mathrm{sire}}_{i}^{2}+{\mathrm{dam}}_{i}^{2}}{2}$ (VanRaden and Sanders 2003) wherein sire_*i*_ and dam_*i*_ are the proportion of breed *i* in the sire and dam, respectively.

#### Production data

##### Feed intake

Dry Matter Intake measurements taken within ±30 d of methane emission measurements were available on 568 animals across both the growing animals and ewe datasets. Dry matter intake was measured using two separate techniques, for ewes all DMI was recorded while grazing pasture outdoors (*n* = 460 records) and for growing animals all DMI records were measured while housed indoors during the winter period (*n* = 180 records). Outdoor grazing DMI was determined using the n-alkane technique on ewes only, as described by Mayes et al. (1986) and validated for Irish grazing conditions by McGovern et al. (2021). Animals had ad libitum access to perennial ryegrass pasture. Pre-grazing herbage mass ranged from 1,200 to 1,500 kg dry matter (DM) per hectare and was measured using a rising plate meter. Each animal was administered a C32-alkane bolus over 11 consecutive days, with faecal samples collected from days 7 to 12. Herbage samples were also collected, and the ratio of herbage C33 alkane to dosed C32 alkane was used to estimate daily DMI (expressed as kg/DM). Indoor DMI measurements were obtained for growing animals only using a weigh-back method. Animals were individually penned and offered grass silage ad libitum over a 12-day measurement period. The total amount of silage offered and daily refusals were recorded each morning, and DMI was calculated as the difference between feed offered and refusals. For the indoor period, DMI was averaged across the full 12-day measurement phase to generate a single intake value per animal. Across both the indoor and outdoor DMI datasets, DMI records more than ± 3 SD from the mean were discarded. The final feed intake dataset consisted of 270 ewes with 460 DMI records measured at pasture and 180 growing animals with 180 DMI records measured indoors.

##### Average daily gain

As part of the Sheep Ireland national breeding programme body weight is collected at regular intervals within the first year of an animal’s life, thereby enabling the calculation of individual animal growth rates across defined time periods. Average daily gain (ADG) was calculated using a linear regression model for each growing animal with at least two weight measurements both collected within 120 d before and after the methane measurement. Only ADG records between 150 and 350 g d^−1^ were retained (*n* = 2,459) (Table 1).

##### Computed tomography

Computed tomography scan measurements, as described by Clelland et al. (2014), were available on 756 growing animals with methane measurements (Table 3). Only CT measurements within 3 d of methane measurement were used, and only values ± 3 SD from the trait mean were retained for analysis resulting in the retention of 719 animals with CT records. Traits available from the CT measurements included fat mass (kg), muscle mass (kg), predicted kill-out percentage (KO%), rumen volume (litres) and the muscle-to-fat ratio (M: F). Fat and muscle mass were calculated as the total weight (kg) of respective tissues segmented from the CT scan. Kill-out percentage (KO%) was defined as the total tissue weight (fat + muscle + bone) divided by the body weight. Rumen volume (litres) represented the combined volume of the rumen and reticulum, and the muscle-to-fat ratio (M: F) was calculated as muscle weight divided by fat weight.

**Table 3:** Number of records, number of animals, mean (standard deviation in parenthesis), trait range and coefficient of variation (CV) for computed topography (CT) traits in growing animals.

Metric^1^	No. recs	No. ani	μ (SD)	Range	CV
**Fat mass (kg)**	1,104	756	3.75 (2.12)	0.48–12.09	56.53
**Muscle Mass (kg)**	1,104	756	12.79 (3.39)	4.81–23.65	26.51
**KO%**	1,104	756	0.45 (0.04)	0.35–0.59	8.89
**M: F**	1,104	756	3.93 (1.26)	1.49–10.14	32.06
**Rumen Volume (litres)**	1,104	756	6.56 (1.52)	2.33–12.27	23.17

1 KO%: kill-out percentage, M: F: Muscle mass to fat mass ratio.

##### Slaughter

Slaughter data, including date of slaughter, carcass weight, carcass conformation, carcass fat and days to slaughter (number of days from birth to slaughter), were also available. Only animals with a cold carcass weight, measured, on average, 1-hour post-slaughter, ranging between 15 and 26 kg were considered. In addition, only animals slaughtered between 100 and 450 d of age and a slaughter record within 30 d of the gaseous emission measurement were retained. For animals that had more than one methane measurement in this period, the closest measurement to slaughter date was retained. Following all edits 607, animals with carcass weights were retained.

##### Other traits

Other production traits available on animals included weaning weight (kg), defined as the lamb’s weight at the time of weaning, and the number of lambs born, representing the total number of lambs born (alive and dead) per lambing event. Total litter birth weight (kg) referred to the combined birth weight of a litter per lambing event, while total litter weaning weight (kg) captured the combined weaning weights of all lambs reared and weaned per ewe. Ewe body condition score (BCS) was assessed on a five-point scale (Jefferies 1961) in increments of one. Ewe efficiency was calculated as ewe’s weight at mating divided by the total litter weaning weight.

### Methane metrics

In total, twenty methane metrics were derived, categorized into one daily production trait, twelve ratio-based traits, and seven residual traits, with each trait generated separately for growing animals and ewes (where appropriate).

The daily production trait refers to the absolute methane output (g d^−1^) as measured directly using PACs, which estimate daily methane emissions based on gas concentrations recorded during a 50-minute spot sampling period under controlled conditions (O’Connor et al. 2021).

#### Ratio traits

Twelve ratio traits were derived for growing animals and three for ewes. Methane intensity was defined in eight separate ways: 1. per kg of body weight (MI_BW_), 2. per kg of metabolic body weight (MI_MBW_), 3. per kg of ADG (growing animals only; MI_ADG_), 4. per kg of carcass weight (growing animals only; MI_CW_) 5. per kg of muscle mass (growing animals only; MI_MM_), 6. per kg of fat mass (growing animals only; MI_FM_), 7. per unit of predicted kill-out percentage (growing animals only; MI_KO_), and finally, 8. per unit of muscle to fat ratio (growing animals only; MI_M: F_):


(1) $${\text{MI}}_{\text{BW}}\;=\;\frac{Methane\;production\;}{Body\;weight}$$

(2) $${\text{MI}}_{\text{MBW}}=\frac{Methane\;production\;}{Metab\text{o}lic\;body\;weight}$$

(3) $${\text{MI}}_{\text{ADG}}=\frac{Methane\;production}{ADG}$$

(4) $${\text{MI}}_{\text{CW}}=\frac{Methane\;production}{Carcass\;weight}$$

(5) $${\text{MI}}_{\text{MM}}=\frac{Methane\;production}{Muscle\;mass}$$

(6) $${\text{MI}}_{\text{FM}}=\frac{Methane\;production}{Fat\;mass}$$

(7) $${\text{MI}}_{\text{KO}}=\frac{Methane\;production}{Kill-out\;percentage}$$

(8) $${\text{MI}}_{\text{M}:\text{F}}=\frac{Methane\;production}{Muscle\;to\;fat\;ratio}$$

where methane production is the daily methane output calculated per animal in grams per day, body weight is the individual animal body weight measurement that coincided with the gaseous emissions measurements in kilograms, metabolic body weight is body weight^0.75^ at the point of gaseous emission measurement in kilograms, ADG is the average daily gain of the animal coinciding with the timing of methane measurement in grams per day, carcass weight is the cold carcass weight in kilograms, muscle and fat mass in kilograms were derived from CT scan segmentation of respective tissue types at methane measurement, predicted CT kill-out percentage was total tissue mass (fat, muscle, bone) divided by body weight (%), and muscle to fat ratio (in kilograms) was muscle weight divided by fat weight.

Methane per liter of rumen volume (MI_rumen_) was measured in growing animals only and was calculated as:


(9) $${\text{MI}}_{\text{Rumen}}=\frac{Methane\;production}{Rumen\;Volume}$$

where rumen volume is the rumen volume of the animal measured through CT scan.

Methane yield (MY) was defined as:


(10) $$MY=\frac{\mathrm{Methane}\;\mathrm{Production}\;}{\mathrm{DMI}\;}$$

where DMI is the dry matter intake measured in grams per day.

Two carbon dioxide-based traits were derived for both growing animals and ewes. The ratio of methane to total gaseous carbon output was defined as:


(11) $${CH}_{4}/{{(CH}_{4}+CO}_{2})=\frac{\mathrm{Methane}\;\mathrm{Production}\;}{\mathrm{Methane}\;\mathrm{Production}+{CO}_{2}\;\mathrm{Production}\;}$$

where methane and carbon dioxide production were expressed in grams per day and measured concurrently in PACs.

CO_2_ Yield was defined as:


(12) $${CO}_{2}Y\mathrm{ield}=\frac{{CO}_{2}P\mathrm{roduction}\;}{\mathrm{DMI}\;}$$

where carbon dioxide production is expressed in grams per day and DMI is dry matter intake measured in grams per day_._

#### Residual traits

A total of seven distinct residual methane traits were derived whilst adjusting for specific production traits. For the calculation of all residual traits, daily methane output was included as the dependent variable and a series of fixed and random effects were fitted to a linear mixed model in R (4.2.3) (R Core Team 2024) using lme4 (1.132) (Bates et al. 2015).

Residual methane traits were derived using linear mixed models fitted separately for growing animals and ewes. For growing animals, the general model was:


$$Y_{i}=\mu +G_{j}+A_{k}+\beta_{1}X_{1i}+\beta_{2}X_{2i}+\beta_{3}X_{3i}+\beta_{4}X_{4i}+\beta_{5}X_{5i}+\beta_{6}X_{6i}+\sum_{b=1}^{B-1}\beta_{b}B_{bi}+PT(s{)}_{i}+e_{i}$$

where $Y_{i}$ was daily methane emissions (g d^−1^), μ  was the overall mean, $G_{j}$ was the random contemporary group effect of flock by measurement date by PAC run, and $A_{k}$ was the random animal effect. Fixed effects included sex (X_1_; male or female), birth litter size (X_2_; 1, 2, ≥3), rearing litter size (X_3_; 1, 2, ≥3), age in months (X_4_; 4–19 months), and the covariates of recombination loss (X_5_; 0.00–0.73) and heterosis (X_6_; 0.00–1.00). Breed proportions ($\sum_{b=1}^{B-1}\beta_{b}B_{b}$) were fitted as fixed effects for Belclare, Blackface Mountain, Charollais, Cheviot, Lleyn, Texel, and Other, with Suffolk omitted as the reference breed. The term PT(s) represents one production trait or a combination of production traits included in each model and $e_{i}$ was the residual term.

Seven residual methane traits were derived for growing animals: methane adjusted for body weight (RM_BW_), metabolic body weight (RMT_MBW_), dry matter intake (RMT_DMI_), metabolic body weight and dry matter intake (RMT_MBW+DMI_), average daily gain (RMT_ADG_), carcass weight (RMT_CW_), and metabolic body weight and average daily gain (RMT_MBW+ADG_).

For ewes, the same modeling framework was used, with modifications to reflect ewe-specific biology. Fixed effects included age in years (X_1_; 1 to ≥7 years), lactation status (X_2_; lactating or dry), birth litter size (X_3_), rearing litter size (X_4_), and the covariates of recombination loss (X_5_; 0.00–0.82) and heterosis (X_6_; 0.00–1.00). Breed effects included Belclare, Charollais, Cheviot, Lleyn, Texel, Vendeen, and Other, with Suffolk again used as the reference breed.

Four residual methane traits were derived for ewes: methane adjusted for body weight (RMT_BW_), metabolic body weight (RMT_MBW_), dry matter intake (RMT_DMI_), and metabolic body weight and dry matter intake (RMT_MBW+DMI_).

### Statistical analysis

Approximately 28% of both growing animals (979) and ewes (1,096) had repeated methane and live-weight records, while repeated dry matter intake measurements were available for 90% of ewes that had DMI records (242). Therefore, the repeatability (*R*) of methane, body weight, and DMI (ewes only) was calculated using a linear mixed model as:


$$R=\;\frac{\sigma_{A}^{2}}{\sigma_{A}^{2}+\;\sigma_{e}^{2}}$$

where $\sigma_{A}^{2}\;$is the between-animal variance and $\sigma_{e}^{2}$ is the residual variance.

Correlations were calculated between all methane metrics in both growing animals and ewes. In addition, correlations were calculated between methane metrics and key production traits. For growing animals, these included body weight, metabolic body weight, dry matter intake (DMI), average daily gain (ADG), carcass weight, rumen volume, days to slaughter, kill-out percentage, and muscle-to-fat ratio. For ewes, traits included weaning weight, number of lambs born, total birth weight of litter, total weaning weight of litter, body condition score (BCS), and an ewe efficiency trait (mating weight divided by total litter weaning weight).

Estimated marginal means were calculated in growing animals from the residual methane models using the eemeans package (Lenth 2023). Comparisons were made for sex while holding continuous covariates at their sample means and evaluating categorical covariates at their reference levels; population-average random effect means were included.

To evaluate how different methane metrics influence animal ranking, the most efficient 25% of animals were identified under four methane metrics, daily methane, MI_MBW_, MY, and RMT_MBW+DMI_. Only animals with complete data for all four metrics were included in the analysis. For each animal, the most efficient record, defined as the measurement with the lowest value for each metric, was identified and used for ranking. This resulted in a final comparison set of 45 growing animals and 68 ewes. Mean performance across key traits such as DMI, methane output, body weight, and carcass characteristics was compared across the resulting efficiency groups.

## Results

The mean, standard deviation, range, and coefficient of variation for both growing animals and ewes across production traits and methane metrics are provided in Tables 1 and 2, respectively. Across contemporary groups (i.e. date of measurement, flock, and PAC run number), 342 contained females only, 47 contained males only, and 244 contained both males and females.

The main breeds (average breed proportions in parentheses) represented in the dataset were Texel (30%), Suffolk (22%), and Belclare (15%). The average (standard deviation in parentheses) age at methane measurement in growing animals was 7.34 (0.40) months; for ewes the average age at measurement was 3.87 (1.62) years. For growing animals, the average DMI was 1.53 (0.60) kg d^−1^ and an average body weight of 47.69 (11.98) kg. The average ewe DMI was 1.86 (0.72) kg d^−1^, with an average body weight of 74.81 (9.26) kg. The mean ADG, days to slaughter and carcass weight in growing animals were 0.21 (0.05) kg d^−1^, 189.00 (12.64) days and 20.13 (SD = 2.14) kg, respectively. Across both growing animals and ewes, residual methane production traits showed substantial individual variability (Tables 1 and 2). In growing animals the standard deviation of RMT_MBW_ was 1.99 g d^−1^ and ranged from −10.36 to +10.12 g d^−1^. For ewes, the standard deviation for RMT_MBW_ was 3.07 g d^−1^, with a range of −16.62 g d^−1^ to13.46 g d^−1^.

The repeatability of daily methane emissions was moderate in both growing animals (0.26 ± 0.02) and ewes (0.34 ± 0.02). In contrast, body weight exhibited high repeatability in both growing animals (0.73 ± 0.01) and ewes (0.69 ± 0.01). The repeatability of dry matter intake in ewes was intermediate (0.51 ± 0.04).

### Sex and breed effects

For growing animals, estimated marginal means from the residual methane models were used to compare sexes; results showed that males consistently produced more methane in comparison to females, with differences ranging from 0.55 (RMT_MBW_) g d^−1^ to 1.36 (RMT_CW_) g d^−1^ (*P* < 0.001; Table 4).

**Table 4:** Marginal means (standard error in parentheses) for males and females within growing animals for residual methane traits.

Metric^1^	Female	Male	*P*-value
**RMT_BW_ (g d^−1^)**	13.81 (0.17)	14.57 (0.20)	<0.01
**RMT_MBW_ (g d^−1^)**	13.82 (0.17)	14.57 (0.20)	<0.01
**RMT_ADG_ (g d^−1^)**	13.98 (0.24)	14.78 (0.24)	<0.01
**RMT_CW_ (g d^−1^)**	14.66 (0.34)	15.99 (0.33)	<0.01
**RMT_MBW+ADG_ (g d^−1^)**	14.10 (0.23)	14.66 (0.23)	<0.01

1 RMT_BW_: residual methane adjusted for body weight, RMT_MBW:_ residual methane adjusted for metabolic body weight, RMT_ADG_: residual methane adjusted for average daily gain, RMT_CW_: residual methane adjusted for carcass weight, RMT_MBW+ADG_: residual methane adjusted for metabolic body weight and average daily gain.

In growing animals, a 1% increase in Charollais breed proportion (relative to Suffolk) was associated with a significant reduction in methane production of −0.021 g d^−1^ (S.E. = 0.006; *P* < 0.001) for the RMT_MBW+ADG_ trait model. A similar pattern was observed for the RMT_CW_ model, where each 1% increase in Charollais breed proportion corresponded to a reduction of −0.018 g d^−1^ (S.E. = 0.006; *P* < 0.01). In ewes, when either MBW or BW was accounted for in the residual models, Belclare and Cheviot breed proportions were associated with significantly lower methane production relative to Suffolk. Specifically, in the RMT_MBW+DMI_ model, each 1% increase in Belclare and Cheviot breed proportion corresponded to reductions of −0.022 g d^−1^ (S.E. = 0.005; *P* < 0.001) and −0.044 g d^−1^ (S.E. = 0.009; *P* < 0.001), respectively.

### Correlations between alternative methane metrics

#### Growing animals

**Figure 1:**
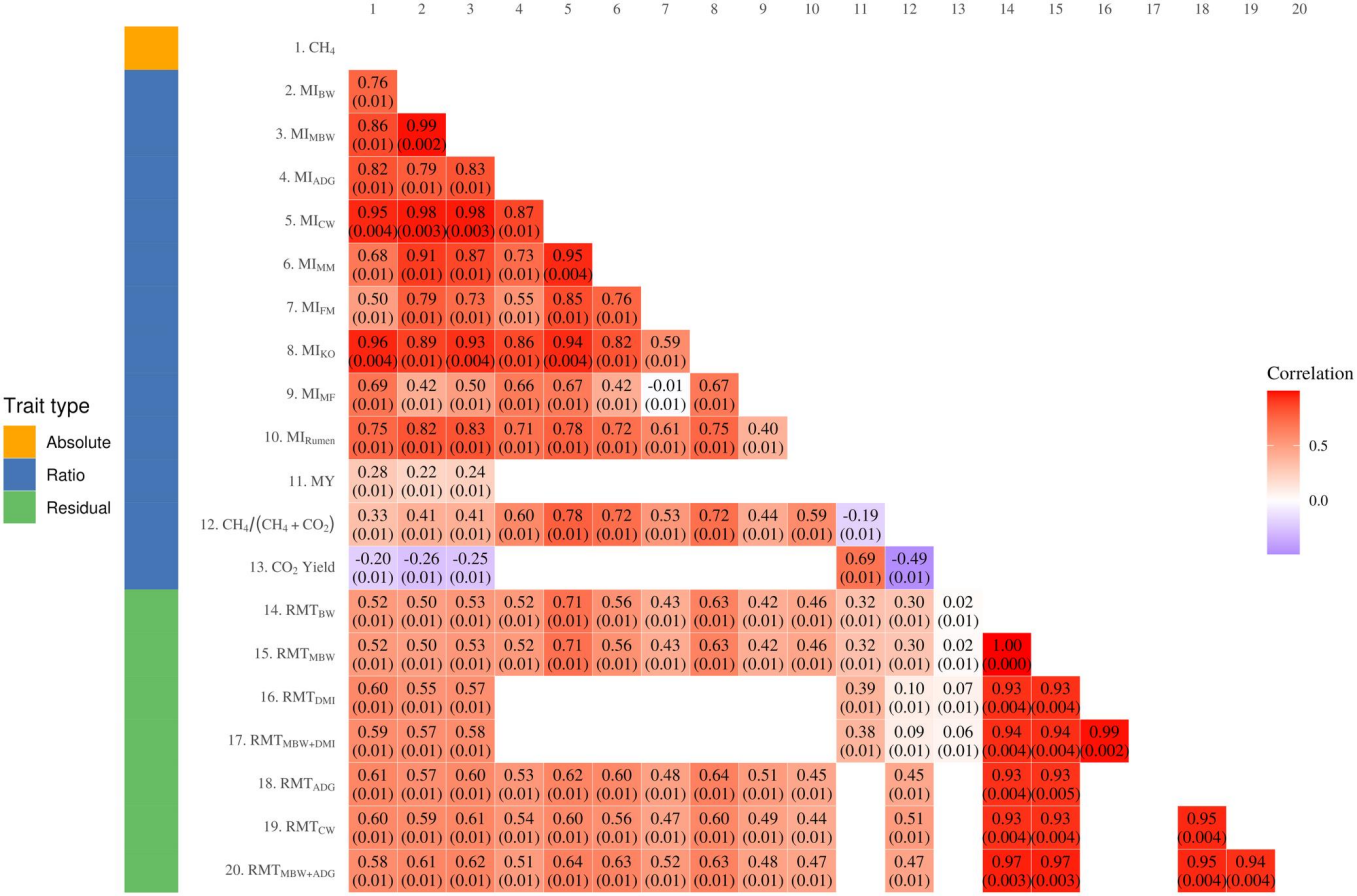
Correlations among absolute, ratio and residual methane metrics in growing animals. CH_4_, daily methane production (g d^−1^); CH_4_/(CH_4_ + CO_2_), methane production as a proportion of methane and carbon dioxide production; CO_2_ Yield, carbon dioxide per kg of DMI; MI_ADG_, methane per kg average daily gain; MI_BW_, methane per kg body weight; MI_CW_, methane per kg carcass weight; MI_FM_, methane per kg fat mass; MI_FO_, methane per unit predicted kill-out percentage; MI_MBW_, methane per kg metabolic body weight; MI_MF_, methane per unit muscle-to-fat ratio; MI_MM_, methane per kg muscle mass; MI_RUMEN_, methane per litre of rumen volume; MY, methane yield per kg dry matter intake; RMT_ADG_, residual methane adjusted for average daily gain (g d^−1^); RMT_BW_, residual methane adjusted for body weight (g d^−1^); RMT_CW_, residual methane adjusted for carcass weight (g d^−1^); RMT_DMI_, residual methane adjusted for dry matter intake (g d^−1^); RMT_MBW_, residual methane adjusted for metabolic body weight (g d^−1^); RMT_MBW+ADG_, residual methane adjusted for metabolic body weight and average daily gain (g d^−1^); RMT_MBW+DMI_, residual methane adjusted for both dry matter intake and metabolic body weight (g d^−1^).

Daily methane emissions showed moderate to strong positive correlations with all methane intensity metrics and ranged from 0.51 ± 0.01 (MI_FM_) to 0.95 ± 0.004 (MI_CW_ and MI_KO_) (Figure 1). The correlation between daily methane emissions and MY was 0.28 ± 0.01. Moderate correlations were recorded between daily methane emissions and all residual traits and were similar to each other (0.52–0.60; Figure 1). Among methane ratio metrics, methane intensity metrics showed moderate to strong agreement with each other (0.46–0.99; Figure 1). In contrast, MY had weak correlations with both MI_MBW_ and MI_BW_ (0.24 ± 0.01 and 0.22 ± 0.01, respectively). All CT-based ratio traits showed strong positive correlations amongst each other and with other intensity traits. Strong correlations were estimated between MI_KO_ and both MI_MBW_ and MI_CW_ (0.93 ± 0.004 and 0.95 ± 0.004, respectively). CH_4_/(CH_4_ + CO_2_) showed a moderate positive correlation with daily methane emissions (0.33 ± 0.01) and a strong positive correlation with MI_CW_ (0.78 ± 0.01), while exhibiting a moderate negative correlation with CO_2_ yield (−0.49 ± 0.01; Figure 1). In contrast, CO_2_ yield was weakly negatively correlated with daily methane emissions (−0.20 ± 0.01) but showed a strong positive association with methane yield (0.69 ± 0.01; Figure 1).

Residual methane traits were all moderately to strongly inter-correlated and ranged from 0.93 ± 0.004 (RMT_DMI_ and RMT_MBW_) to 0.99 ± 0.004 (RMT_MBW_ and RMT_BW_). The correlations between methane ratio and residual methane traits, were generally moderate to strong (range 0.42–0.71), with the exception of the correlations between all methane residual traits and MY and CO_2_ based traits which were weaker overall (Figure 1).

#### Ewes

**Figure 2:**
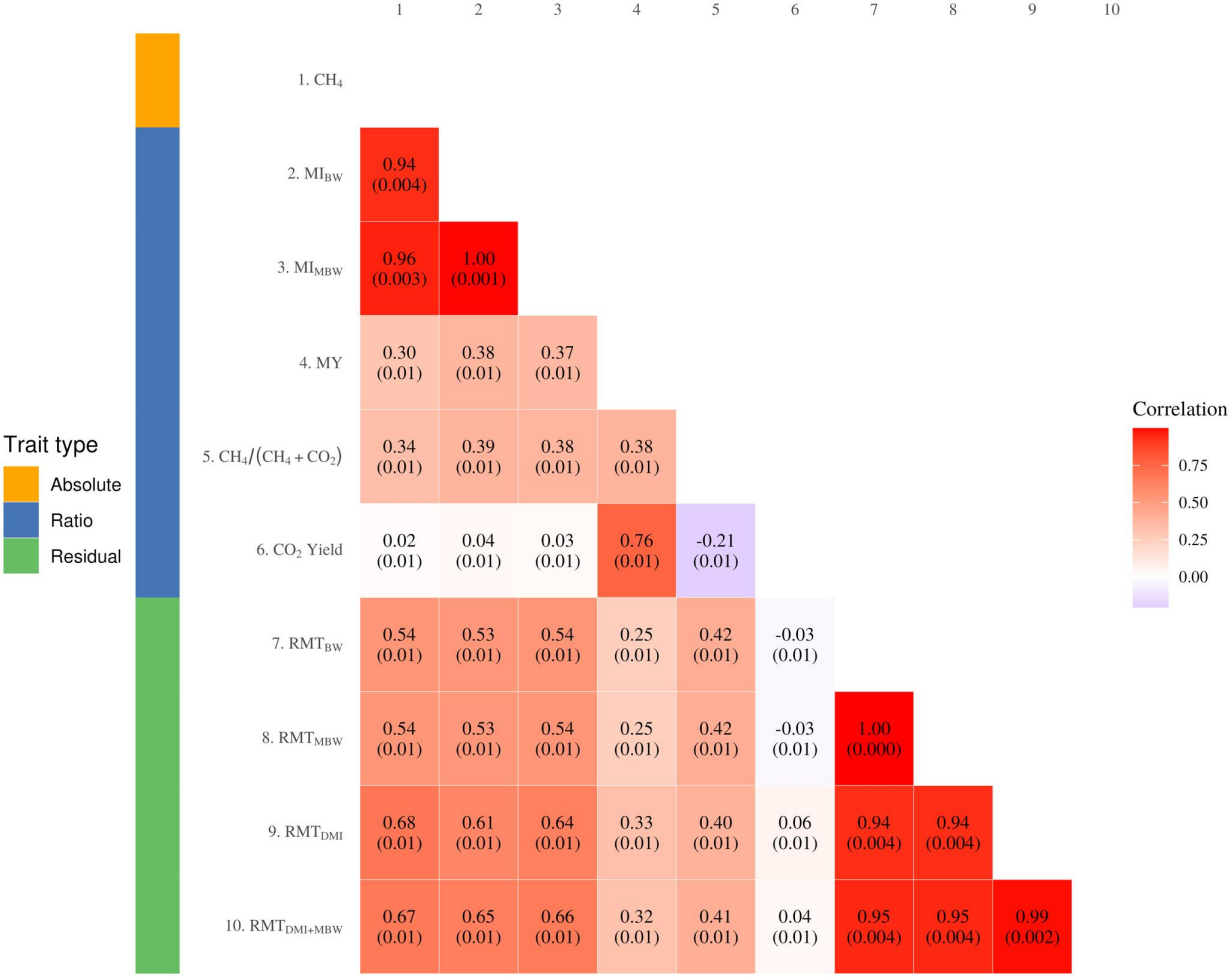
Correlations among absolute, ratio and residual methane metrics in ewes. CH_4_/(CH_4_ + CO_2_), methane production as a proportion of methane and carbon dioxide production; CH_4_, daily methane production (g d^−1^); CO_2_ Yield, carbon dioxide per kg of DMI; MI_BW_, methane per kg body weight; MI_MBW_, methane per kg metabolic body weight; MY, methane yield per kg dry matter intake; RMT_BW_, residual methane adjusted for body weight (g d^−1^) ; RMT_DMI_, residual methane adjusted for dry matter intake (g d^−1^); RMT_MBW_, residual methane adjusted for metabolic body weight (g d^−1^); RMT_MBW+DMI_, residual methane adjusted for both dry matter intake and metabolic body weight (g d^−1^).

Overall, the trends among the methane metrics were similar in ewes to those observed in the growing animal population. A close to unity correlation was recorded between daily methane emissions and MI_BW_ (0.94 ± 0.004) and MI_MBW_ (0.96 ± 0.003), while the correlation between daily methane emissions and MY was moderate (0.30 ± 0.01; Figure 2). The correlations between daily methane emissions and the methane residual traits ranged from 0.54 ± 0.01 (daily methane and RMT_MBW_) to 0.68 ± 0.01 (daily methane and RMT_DMI_). Among the ratio metrics, MI_MBW_ and MI_BW_ were almost perfectly correlated (0.99 ± 0.003). The CH_4_/CH_4_ + CO_2_ ratio trait showed a moderate positive correlation with daily methane emissions (0.34 ± 0.01) and a weak negative correlation with CO_2_ yield (−0.21 ± 0.01; Figure 1). In contrast, CO_2_ yield was weakly associated with daily methane emissions (0.02 ± 0.01) but showed a strong positive correlation with methane yield (0.76 ± 0.01). Close to unity correlations were also observed among the ewe residual methane traits (range 0.94–0.99; Figure 2). Moderate correlations were observed between MY and both MI_MBW_ and MI_BW_ (0.37 ± 0.01–0.38 ± 0.01; Figure 2). With the exception of MY and CO_2_ Yield, the correlation between the methane ratio and residual traits were moderate (0.53–0.66).

### Correlations between production traits and methane metrics

#### Growing animals

Animals with higher daily emissions tended to have higher body weight (0.30 ± 0.01), however, this association was not observed in methane intensity metrics accounting for body weight (i.e. MI_BW_ and MI_MBW_). Dry matter intake was also positively correlated with daily emissions (0.42 ± 0.04), and this persisted in the methane intensity metric MI_MBW_ and MI_BW_ (*r* = 0.44 ± 0.03 and 0.44 ± 0.03, respectively). Growth traits followed slightly more nuanced patterns; days to slaughter (DTS) showed positive correlations with daily methane emissions (0.22 ± 0.04), suggesting that higher-emitting animals take longer to finish. Positive correlations were observed between DTS and almost all methane ratio metrics (Table 5). Carcass weight was negatively correlated with daily methane emissions (−0.15 ± 0.03) and also intensity traits (*r* = −0.26 to −0.12; Table 5). The CH_4_/(CH_4_ + CO_2_) ratio exhibited generally weak associations with production traits, with the strongest positive correlations observed for dry matter intake (0.39 ± 0.04) and days to slaughter (DTS; 0.21 ± 0.04; Table 5). In contrast, CO_2_ yield was strongly negatively correlated with DMI (−0.81 ± 0.02) and showed a moderate positive correlation with carcass weight (0.56 ± 0.10; Table 5).

**Table 5.** Correlations between methane metrics and production traits in growing animals.

Metric	Trait^1^	Body weight (kg)	Rumen volume (l)	Dry matter intake (kg d^−1^)	Weaning weight (kg)	Carcass weight (kg)	Average daily gain (kg d^−1^)	Days to slaughter (days)	M: F	KO (%)
**Absolute**	Methane (g d^−1^)	0.30 (0.01)	0.31 (0.03)	0.42 (0.04)	0.07 (0.01)	−0.14 (0.03)	0.09 (0.02)	0.22 (0.04)	−0.11 (0.03)	0.25 (0.03)
**Ratio**	MI_BW_	−0.37 (0.01)	−0.10 (0.03)	0.44 (0.03)	−0.25 (0.01)	−0.26 (0.02)	−0.10 (0.02)	0.20 (0.04)	0.25 (0.03)	0.00
	MI_MBW_	−0.21 (0.01)	NA	0.44 (0.03)	−0.18 (0.01)	−0.24 (0.02)	−0.05 (0.02)	0.21 (0.04)	0.17 (0.03)	0.00
	MI_ADG_	0.12 (0.02)	0.14 (0.04)	NA	−0.09 (0.02)	−0.12 (0.03)	−0.45 (0.02)	0.30 (0.04)	0.00	0.00
	MI_CW_	0.15 (0.04)	0.15 (0.04)	NA	0.00	−0.25 (0.04)	0.00	0.22 (0.04)	0.13 (0.04)	−0.17 (0.04)
	MI_MM_	−0.25 (0.03)	−0.10 (0.03)	NA	−0.11 (0.04)	−0.18 (0.04)	0.16 (0.04)	0.00	0.00	−0.39 (0.03)
	MI_FM_	−0.39	−0.21	NA	−0.30	−0.25	0.10	0.13	0.65	−0.27
	MI_KO_	0.24 (0.03)	0.22 (0.03)	NA	0.00	0.00	0.19 (0.04)	0.00	0.00	0.00
	MI_M: F_	0.58 (0.02)	0.37 (0.03)	NA	0.24 (0.03)	0.19 (0.04)	0.22 (0.04)	0.00	−0.60 (0.02)	0.12 (0.03)
	MI_Rumen_	NA	−0.38 (0.03)	NA	−0.13 (0.04)	0.00	0.16 (0.05)	0.18 (0.04)	0.17 (0.04)	0.00
	MY	0.16 (0.04)	NA	−0.69 (0.02)	0.00	0.55 (0.04)	NA	NA	NA	NA
	CO_2_ Yield	0.20 (0.04)	NA	0.39 (0.02)	0.17 (0.04)	0.56 (0.13)	NA	NA	NA	NA
	CH_4_/(CO_2_ + CH_4_)	−0.12 (0.01)	0.00	0.39 (0.04)	−0.51 (0.01)	−0.09 (0.03)	−0.15 (0.02)	0.21 (0.04)	0.10 (0.03)	−0.06 (0.03)
**Residual**	RMT_BW_	0.00	0.11 (0.03)	0.00	0.00	0.00	0.00	0.00	0.00	−0.06 (0.03)
	RMT_MBW_	NA	0.11 (0.03)	0.00	0.00	0.00	0.00	0.00	0.00	−0.07 (0.03)
	RMT_DMI_	0.12 (0.04)	NA	0.00	0.00	0.00	NA	NA	NA	NA
	RMT_MBW+DMI_	NA	NA	0.00	0.00	0.00	NA	NA	NA	NA
	RMT_ADG_	0.15 (0.02)	0.19 (0.04)	NA	0.11 (0.02)	0.00	0.00	0.12 (0.05)	0.00	0.00
	RMT_CW_	0.16 (0.03)	0.20 (0.04)	NA	0.10 (0.03)	0.00	0.12 (0.04)	0.00	0.00	0.00
	RMT_MBW+ADG_	0.00	0.15 (0.04)	NA	0.00	0.00	0.00	0.00	0.00	0.00

1 MI_BW_: methane per kg body weight, MI_MBW_: methane per kg metabolic body weight, MI_ADG_: methane per kg of average daily gain, MI_CW_: methane per kg carcass weight, MI_MM_: methane per kg muscle mass, MI_FM_: methane per kg fat mass, MI_KO_: methane per unit KO%: MI_M: F_: methane per muscle-to-fat ratio: MI_Rumen_: methane per litre of rumen volume, MY: methane yield per kg dry matter intake, CH_4_/(CH_4_ + CO_2_): methane production as a proportion of methane and carbon dioxide production, CO2 Yield: carbon dioxide per kg of dry matter intake, RMT_BW_: residual methane adjusted for body weight (g d^−1^), RMT_MBW_: residual methane adjusted for metabolic body weight (g d^−1^), RMT_DMI_: residual methane adjusted for dry matter intake (g d^−1^), RMT_MBW+DMI_: residual methane adjusted for metabolic body weight and dry matter intake (g d^−1^), RMT_ADG_: residual methane adjusted for average daily gain (g d^−1^), RMT_CW_: residual methane adjusted for carcass weight (g d^−1^), RMT_MBW+ADG_: residual methane adjusted for metabolic body weight and average daily gain (g d^−1^). NAs indicate lack of overlapping data.

#### Ewes

Larger ewes with higher intake emitted more absolute methane, which was reflected in the weak positive correlations estimated between absolute methane and both body weight and DMI (0.17 ± 0.01 and 0.24 ± 0.03, respectively). Weak to no correlations were estimated between methane metrics and other key performance traits such as number of lambs born, litter weaning weight, ewe efficiency, and BCS (−0.07 to 0.14).

Across both growing animals and ewes, residual methane traits, showed no correlation with covariates (i.e. weight and DMI etc.), and close to zero correlations with any traits that were not explicitly included in the models, such as rumen volume.

### Effects of selecting based on different metrics

The top 25% of efficient growing animals, as determined by MY on average were associated with a DMI of 1.89 kg d^−1^, which was significantly higher than the 25% most efficient growing animals based on daily methane (1.22 kg d^−1^) or MI_MBW_ (1.18 kg d^−1^; Table 7; *P* < 0.001). Growing animals ranked by RMT_DMI+MBW_ (1.75 kg d^−1^) also had a greater dry matter intake than daily methane and MI_MBW_ ranked animals (*P* < 0.001). The highest daily methane emissions were observed when growing animals were selected based on RMT_DMI+MBW_ (9.90 g d^−1^), followed by MI_MBW_ (6.04 g d^−1^; *P* < 0.0001), MY (7.56 g d^−1^; *P* < 0.001), and daily methane emissions (5.96 g d^−1^; *P* < 0.0001). Growing animals ranked based on the different metrics did not differ significantly between groups in terms of body weight or weaning weight.

**Table 7.** Mean dry matter intake, daily methane emissions, body weight and weaning weight for the most efficient 25% of growing animals based on four methane efficiency metrics^1^.

Metric	Dry matter intake (g d^−1^)	Methane (g d^−1^)	Body weight (kg)	Weaning weight (kg)
**Methane (g d^−1^)**	1.22 (0.64)^a^	5.96 (1.00)^a^	47.96 (6.24)^a^	35.29 (4.32)^a^
**MI_MBW_**	1.18 (0.61)^a^	6.04 (1.13)^a^	49.24 (6.70)^a^	35.79 (4.60)^a^
**MY**	1.88 (0.39)^b^	7.56 (2.03)^b^	47.41 (6.40)^a^	34.08 (4.19)^a^
**RMT_DMI+MBW_ (g d^−1^)**	1.75 (0.58)^b^	9.90 (4.06)^c^	48.95 (6.00)^a^	35.03 (4.58)^a^

1 MI_MBW_: methane per kg of metabolic body weight, MY: methane yield per kg dry matter intake, RMT_DMI+MBW_: residual methane adjusted for dry matter intake and metabolic body weight.

a,b,c Values within a column with different superscripts differ significantly (*P* < 0.05).

Similar to growing animals, ewes ranked as the 25% most efficient by MY had substantially higher average DMI (2.66 kg d^−1^) than those ranked by daily methane (1.66 kg d^−1^), MI_MBW_ (1.70 kg d^−1^), or RMT_MBW+DMI_ (1.82 kg d^−1^) (Table 8; *P* < 0.001). In terms of daily methane output, ewes ranked by MY (16.71 g d^−1^) and RMT_MBW+DMI_ (16.67 g d^−1^) had significantly higher emissions than those selected by MI_MBW_ (13.44 g d^−1^; *P* < 0.001) and daily emissions (13.23 g d^−1^; *P* < 0.001). Irrespective of the methane metric used to rank the top 25% of ewes, performance did not differ across body weight, number of lambs born, or number of lambs reared (Table 8).

**Table 8.** Mean dry matter intake, daily methane emissions, body weight, number of lambs born and rearing litter size for the most efficient 25% of ewes based on four methane efficiency metrics^1^.

Metric^1^	Dry matter intake (kg d^−1^)	Methane (g d^−1^)	Body weight (kg)	Number of lambs born	Number of lambs reared
**Methane (g d^−1^)**	1.66 (0.83)^a^	13.23 (2.48)^a^	75.41 (8.68)^a^	1.93 (0.68)^a^	1.74 (0.51)^a^
**MI_MBW_**	1.70 (0.88)^a^	13.44 (2.70)^a^	78.00 (9.12)^a^	1.91 (0.71)^a^	1.72 (0.54)^a^
**MY**	2.66 (0.72)^b^	16.71 (5.16)^b^	78.62 (8.29)^a^	1.88 (0.66)^a^	1.69 (0.50)^a^
**RMT_DMI+MBW_ (g d^−1^)**	1.82 (0.82)^a^	16.67 (4.87)^b^	77.15 (9.87)^a^	1.97 (0.65)^a^	1.75 (0.50)^a^

1 MI_MBW_: methane per kg of metabolic body weight, MY: methane yield per kg dry matter intake, RMT_DMI+MBW_: residual methane adjusted for dry matter intake and metabolic body weight.

a,b Values within a column with different superscripts differ significantly (*P* < 0.05).

## Discussion

There is mounting pressure on agriculture to reduce emissions, particularly enteric methane produced by ruminants, however research on methane traits in sheep remains relativity limited (Jonker et al. 2018; Gebbels et al. 2022; O’Connor et al. 2024). A wide range of methane metrics have been proposed in isolation, including absolute production values, ratio-based metrics, and residual traits (Goopy et al. 2014; Crowley et al. 2024). However, the biological relevance and practical implications of these metrics are not well understood, particularly in pasture-based sheep systems. The present study evaluated and compared a suite of these methane metrics in sheep across life-stages, with the objective of identifying how these definitions differ in their associations with performance traits and with each other. By quantifying these relationships, this study helps clarify the strengths and limitations of each metric and informs future efforts to select or monitor animals based on methane efficiency. Given the diversity of biological and management contexts, it is unlikely that a single methane trait will be optimal for all applications but understanding how trait definitions diverge is an essential step in defining their role in breeding, prediction and mitigation strategies.

### Evidence of animal-level variation in methane emissions

After accounting for key production traits in the residual methane models, moderate correlations remained between residuals and daily methane emissions. This suggests that some animals consistently emit more or less methane than expected, indicating animal-specific variation in methane efficiency. This indication of animal-specific variability in methane efficiency is bolstered by the moderate repeatability estimates observed for methane emissions in the present study, in both growing animals (*R* = 26%) and ewes (*R* = 34%). The consistency of daily methane output within animals suggests that individual animal-level biological factors play a role in individual methane output, rather than the trait being solely environmentally or behaviourally driven. This moderate repeatability is particularly notable given the inherent variability of PACs (Goopy et al. 2011; O' Connor et al. 2024), and the fact that repeated measurements in this study were often taken over extended time periods and different physiological stages. In comparison to the current study, higher repeatability estimates have been previously reported by Jonker et al. (2018) (65–76%) and Robinson et al. (2020) in ewes (78%) in both lambs and ewes, respectively; albeit in both incidences respiration chambers were used across a 14 d interval, which likely contributed to the higher repeatability estimates.

### Methane metrics

The various methane intensity traits, along with methane per rumen volume, all showed strong correlations with daily methane emissions and each other. These findings are consistent with Crowley et al. (2024), who reported similarly strong phenotypic correlations (0.74–0.87) between daily methane production and methane intensity traits in growing cattle. These results suggest that methane intensity metrics investigated in the present study reliably reflect total methane output. However, unlike daily methane emissions, these metrics adjust for key biological differences, mainly body size, thus allowing for a more accurate comparison between animals of differing physiological states, an important consideration for livestock producers who are aiming to improve methane efficiency without compromising growth or carcass traits. While, methane intensity metrics are informative, they remain confounded by factors not explicitly accounted for, such as feed intake, physiological status, genetic effects and microbiome composition (Martínez-Álvaro et al. 2022; Zhang et al. 2023; Bilton et al. 2025). As a result, these metrics do not isolate the animal’s inherent methane-efficiency potential independently of key production traits, thus caution should be taken when interpreting them as direct selection targets.

Unlike methane intensity traits, MY was much less representative of daily methane output across both life stages, highlighted by the weaker correlation reported in the present study between MY and daily methane emissions. Methane yield also displayed a strong negative correlation with its own denominator (DMI; −0.69 ± 0.02 in growing animals and −0.72 ± 0.02 in ewes). While all ratio traits are inherently affected by their denominator (McCaw et al. 2025), this effect was particularly pronounced in the present study due to the higher coefficient of variation (CV) associated with DMI compared to other denominator traits for methane intensity traits such as BW and MBW. This pattern was also observed in data available from Beck et al. (2024) in which beef finishing cattle where DMI showed greater variability than weight. The consistency of the CV reported between both feed intake measurement techniques used in the present study also suggests that the DMI measurement technique was not the reason for high CV. As a result, MY, as quantified in the present study, was disproportionately sensitive to variation in an animal’s intake. Hence, animals with higher DMI often appeared to have lower methane yield, not necessarily because they were more efficient, but because the large variability in DMI suppressed the ratio trait. With this in mind, MY is perhaps best interpreted as a reflection of the effect of diet or intake patterns on methane emissions, and is corroborated by multiple studies that have shown significant diet type and feed pattern effects on methane emissions (Waghorn et al. 2002; Beauchemin and McGinn 2006; Woodmartin et al. 2024). Given this, caution should be exercised for MY to evaluate or rank animal in terms of overall methane efficiency. It is important however, not to conflate the limitations of MY with a lack of relevance of DMI itself; DMI has been shown to have significant positive correlations with methane emissions across life stages (Crowley et al. 2024; Kennedy et al. 2024; O’Connor et al. 2024) a pattern that was also observed in this present study, confirming its biological importance as a standalone trait.

In the present study, carbon dioxide–based metrics captured physiological signals distinct from both methane intensity and methane yield. The CH_4_/(CH_4_ + CO_2_) ratio trait showed only modest correlations with daily methane emissions (0.33 ± 0.01 in growing animals; 0.34 ± 0.01 in ewes) and showed slight negative correlations with methane yield. This supports previous observations in cattle Rosa and Jonker (2022) and emphasises that the CH_4_/(CH_4_ + CO_2_) ratio should not be interpreted as interchangeable with methane yield or methane intensity. In contrast, CO_2_ yield (g CO_2_ kg DMI^−1^) showed stronger and more coherent patterns relative to methane yield, reflecting its closer relationship with intake-driven metabolic processes. Given this relationship, CO_2_ yield may offer potential value for predicting DMI in grazing systems, where intake is otherwise difficult to measure directly. Together, these results suggest that CO_2_ yield and the CH_4_/(CH_4_ + CO_2_) ratio describe different aspects of respiratory gas exchange, with the former more closely linked to intake and the latter reflecting methane as a proportion of total gas output but not a strong indicator of absolute methane production. Residual traits have the ability to capture the remaining variation in a trait after accounting for known environmental and production-related effects (Kennedy et al. 1993; Crowley et al. 2010). Crucially, the methane residual metrics investigated in the current study were moderate to strongly correlated with daily methane emissions, suggesting that they are not composed of random error alone but instead retain meaningful biological interpretation (Donoghue et al. 2016; Crowley et al. 2024). Their consistency and independence of the input factors indicate that they may capture underlying animal-specific factors, such as host genetics or rumen microbiome composition, which have been shown to significantly affect methane emissions (Martínez-Álvaro et al. 2022; Waters et al. 2025).

### Selection outcomes and trait relationships

The variation in animal selection across methane efficiency definitions highlights the impact that metric choice can have on both DMI and methane emission output. Notably, animals ranked as most efficient using MY consistently exhibited higher DMI than those ranked by daily emissions or MI_MBW_, particularly among ewes. This reflects the inherent structure of MY as a ratio trait that can favour high-producing, high-intake animals with proportionally lower emissions (Berry and Crowley 2013; Berry and McCarthy 2021). Conversely, selection based on residual methane (RMT_MBW+DMI_) also identified animals with high DMI and high absolute emissions but whose emissions were lower than expected given their level of production. This reflects the intended purpose of residual traits: to identify animals that emit less methane than predicted based on their size, intake, and growth, rather than favouring smaller or less productive individuals (Hurley et al. 2016). Importantly, absolute methane and MI_MBW_ consistently selected animals with the lowest DMI and daily methane emissions. While this may reduce emissions, these metrics may inadvertently favour smaller or less productive animals, raising questions about their suitability for breeding programs.

Selecting for lower methane emissions must not compromise economically important traits In the present study, lower methane-emitting animals did not exhibit poorer phenotypic performance and, in several cases, were associated with favourable production characteristics. Days to slaughter showed a modest positive correlation with daily methane emissions (0.22 ± 0.04) and MI_MBW_ (0.21 ± 0.02), indicating that high daily emitters may take longer to finish. Carcass weight in growing animals was also negatively correlated with daily methane emissions (−0.14 ± 0.03), this suggests that animals that produce less methane generally have more carcass weight, this remained true when overall body weight was accounted for. Rumen volume showed a moderate positive phenotypic correlation with methane emissions (0.31 ± 0.03), consistent with its role in driving fermentation capacity. However, this association was substantially reduced (partial *r* = 0.07 ± 0.03) when body weight was controlled for. Overall, these results support the conclusion that selecting for methane efficiency may even enhance profitability by favouring animals that convert feed into carcass more effectively and finish earlier.

Ewe methane emissions showed minimal to no correlation with key maternal traits, including NLB (−0.07 ± 0.01), BCS (0.00), total litter weight (0.00), and ewe efficiency (0.00). These patterns were consistent across methane metrics (Table 6), indicating that improvements in environmental efficiency are unlikely to adversely affect reproductive or maternal performance. Although ewe body weight showed a weak positive correlation with daily methane emissions (*r* = 0.17 ± 0.01), this effect is neutralised in methane traits that include weight as a covariate (e.g. MI_MBW_ or RMT_BW_).

**Table 6.** Correlations between methane metrics and production traits in ewes, standard errors in parentheses.

Metric	Trait^1^	Body weight (kg)	Dry matter intake (kg d^−1^)	Number of lambs born	Body condition score (1–5)	Litter weaning weight (kg)	Ewe efficiency
**Absolute**	Methane (g d^−1^)	0.17 (0.01)	0.24 (0.03)	−0.07 (0.01)	0.00	0.00	0.00
**Ratio**	MI_BW_	−0.16 (0.01)	0.10 (0.03)	0.00	−0.17 (0.02)	−0.03 (0.01)	−0.03 (0.01)
	MI_MBW_	−0.07 (0.01)	0.14 (0.01)	−0.03 (0.01)	−0.13 (0.02)	0.00	0.00
	MY	−0.18 (0.03)	−0.72 (0.02)	0.00	0.00	−0.19 (0.03)	0.10 (0.04)
	CH_4_/(CO_2_ + CH_4_)	−0.16 (0.01)	−0.18 (0.03)	0.00	−0.19 (0.02)	0.00	−0.05 (0.01)
	CO_2_ Yield	0.00	−0.66 (0.02)	0.00	0.00	−0.20 (0.03)	0.14 (0.04)
**Residual**	RMT_BW_ (g d^−1^)	0.00	0.00	0.00	−0.09 (0.02)	0.10 (0.01)	−0.10 (0.01)
	RMT_MBW_ (g d^−1^)	0.00	0.00	0.00	−0.09 (0.02)	0.10 (0.01)	−0.10 (0.01)
	RMT_DMI_ (g d^−1^)	0.10 (0.03)	0.00	0.00	0.00	−0.20 (0.03)	0.10 (0.04)
	RMT_MBW+DMI_ (g d^−1^)	0.00	0.00	0.00	0.00	0.00	−0.11 (0.04)

1 MI_BW_: methane per kg of body weight, MI_MBW_: methane per kg of metabolic body weight, MY: methane yield per kg dry matter intake, CH_4_/(CH_4_ + CO_2_): methane production as a proportion of methane and carbon dioxide production, CO_2_ Yield: carbon dioxide per kg of dry matter intake, RMT_BW_: residual methane adjusted for body weight, RMT_MBW_: residual methane adjusted for metabolic body weight, RMT_DMI_: residual methane adjusted for dry matter intake, RMT_MBW+DMI_: residual methane adjusted for metabolic body weight and dry matter intake, Ewe Efficiency: ewe mating weight divided by total litter weight weaned.

## Conclusion

This study highlights the complexity of defining and interpreting alternative methane emissions metrics in sheep and underscores the importance of selecting appropriate metrics based on context and intended application. While absolute and intensity-based traits provide useful insights into total emissions and can account for the size of an animal, they are also confounded by many factors. In the present study, methane yield was disproportionately influenced by DMI variability, and as such, the interpretation of this metric may be more appropriate in the context of dietary comparisons or feedstuff efficiency, rather than as standalone indicators of animal-level methane efficiency. Residual traits offer promise, not necessarily as traits to be selected on directly, but as a means of isolating underlying biological factors such as host genetics or rumen microbiome effects. When incorporating methane into selection programmes, it is best to model absolute methane emissions while accounting for relevant production traits, as ratio traits can be problematic in linear genetic evaluations due to their statistical properties (Berry and Crowley 2013). Therefore, to best support the development of biologically meaningful methane measurement, future studies should consider the inclusion of relevant performance traits, especially DMI, metabolic body weight, average daily gain, carcass weight, and lactation status.

## Abbreviations

ADG: average daily gain
BCS: body condition score
BW: body weight
CH_4_: Methane
CH_4_/(CH_4_ + CO_2_): daily methane as a proportion of daily methane plus daily CO_2_
CO_2_ Yield: CO_2_ per kg dry matter intake
CO_2_: carbon dioxide
CT: computed tomography
CW: carcass weight
DMI: dry matter intake
FM: fat mass
Kg: kilogram
MBW: metabolic body weight
MI_ADG_: methane per kg of average daily gain
MI_BW_: methane per kg of body weight
MI_CW_: methane per kg of carcass weight
MI_FM_: methane per kg of fat mass
MI_KO_: methane per unit predicted KO%
MI_MBW_: methane per kg of metabolic body weight
MI_MF_: methane per unit muscle to fat ratio
MI_MM_: methane per kg of muscle mass
MI_RV_: methane per litre rumen volume
MM: muscle mass
MY: methane yield (methane per kg dry matter intake)
PAC: portable accumulation chambers
RMT_ADG_: residual methane adjusted for average daily gain
RMT_BW_: residual methane adjusted for body weight
RMT_CW_: residual methane adjusted for carcass weight
RMT_DMI_: residual methane adjusted for dry matter intake
RMT_MBW_: residual methane adjusted for metabolic body weight
RMT_MBW+ADG_: residual methane adjusted for metabolic body weight and average daily gain
RMT_MBW+DMI_: residual methane adjusted for metabolic body weight and dry matter intake
